# Supplementary material for: Barriers to widespread adoption of telehealth from physicians’ perspective: A survey in southern Iran
Source: PLoS One. 2025 Jul 21;20(7):e0327095. doi: 10.1371/journal.pone.0327095 (PMC12279122; doi:10.1371/journal.pone.0327095)
Supplement: S1 Appendix — (DOCX) [file pone.0327095.s001.docx]

**Supplementary Appendix 1: Questionnaire**

**Demographic**

Age (by year): Gender:

level of education: Specialist 🞎 General practitioner 🞎

Years of professional experience:

**Barriers to adoption of telehealth from physicians’ perspective**

| **Variables and Items** | | Strongly disagree | Disagree | Neutral | Agree | Strongly agree |
| --- | --- | --- | --- | --- | --- | --- |
| Organizational | Telehealth implementation requires coordination between different parts of the health system. |  |  |  |  |  |
|  | The government does not adequately support the use of telehealth. |  |  |  |  |  |
|  | The university does not adequately support the use of telehealth. |  |  |  |  |  |
|  | The public does not welcome the use of telehealth. |  |  |  |  |  |
|  | It is difficult to issue a license (license) and accreditation for telehealth. |  |  |  |  |  |
|  | There are no regulations for the use of telehealth. |  |  |  |  |  |
|  | There is no appropriate space in hospitals to use this system. |  |  |  |  |  |
|  | The private sector is not willing to provide telehealth services. |  |  |  |  |  |
|  | Appropriate training is not provided for medical staff and patients. |  |  |  |  |  |
|  | There is no technical expertise to support telehealth. |  |  |  |  |  |
|  | Due to the lack of previous experience, there is no possibility of mutual learning and knowledge exchange between health organizations. |  |  |  |  |  |
|  | There is no written plan for structured and widespread use of telehealth in the Ministry of Health (MOH). |  |  |  |  |  |
|  | Issues such as access to technology and health literacy |  |  |  |  |  |
| Financial | There is little resource allocation for telehealth. |  |  |  |  |  |
|  | Insurance companies do not pay for these services. |  |  |  |  |  |
|  | Initial costs (deployment and setup) of the system are high. |  |  |  |  |  |
|  | Ongoing costs of the system are high. |  |  |  |  |  |
|  | Costs to care providers increase. |  |  |  |  |  |
|  | Cost of internet access is high. |  |  |  |  |  |
|  | Evidence regarding its effectiveness or cost-effectiveness is limited. |  |  |  |  |  |
|  | Costs of information exchange are high. |  |  |  |  |  |
| Clinical | Reduces diagnostic accuracy. |  |  |  |  |  |
|  | Reduces quality of care. |  |  |  |  |  |
|  | Increased risk of medication errors. |  |  |  |  |  |
|  | Takes up more time from healthcare professionals. |  |  |  |  |  |
|  | Clinical protocols for patient care in telehealth are absent or unavailable. |  |  |  |  |  |
|  | There are serious concerns about the safety of telehealth. |  |  |  |  |  |
|  | Physical examination is not possible. |  |  |  |  |  |
|  | Data quality is not adequate. |  |  |  |  |  |
|  | Documentation quality is not adequate. |  |  |  |  |  |
|  | Potential for inappropriate interpretation of information. |  |  |  |  |  |
|  | Negatively impacts physician-patient communication. |  |  |  |  |  |
|  | Negatively impacts communication between healthcare professionals. |  |  |  |  |  |
|  | It is not possible to perform different laboratory tests. |  |  |  |  |  |
| Technical | It is difficult to set up software and related programs. |  |  |  |  |  |
|  | Current bandwidth does not allow for stable and uninterrupted communication. |  |  |  |  |  |
|  | Communication is disrupted when multiple people log into the system at the same time. |  |  |  |  |  |
|  | Many people in remote areas do not have access to the Internet. |  |  |  |  |  |
|  | Many people in remote areas do not have access to the necessary device (mobile, tablet or computer). |  |  |  |  |  |
|  | There are technical problems in using related hardware and software. |  |  |  |  |  |
|  | There are problems with Internet interruptions or filtering. |  |  |  |  |  |
|  | Technical standards related to telehealth are low and difficult to implement. |  |  |  |  |  |
|  | It is difficult for patients to connect to the system (complicated or difficult steps during the first registration). |  |  |  |  |  |
|  | Due to the current infrastructure, it is not possible to use peripheral devices (sphygmomanometer, pedometer, etc.). |  |  |  |  |  |
|  | Telehealth cannot be used in all specialties. |  |  |  |  |  |
| Behavioral | Patients prefer face-to-face contact with doctors. |  |  |  |  |  |
|  | The quality of telehealth services is questionable. |  |  |  |  |  |
|  | There is no appropriate culture for using this technology. |  |  |  |  |  |
|  | Doctors resist using this system. |  |  |  |  |  |
|  | Patients resist using this system. |  |  |  |  |  |
|  | Health care workers resist using this system. |  |  |  |  |  |
|  | There is insufficient awareness about this technology and its benefits. |  |  |  |  |  |
| Legal | There are concerns about medical malpractice. |  |  |  |  |  |
|  | There are strict regulatory laws. |  |  |  |  |  |
|  | There are serious concerns about the security of telehealth. |  |  |  |  |  |
|  | There are serious concerns about patient privacy. |  |  |  |  |  |
|  | It is difficult to obtain informed consent in telehealth. |  |  |  |  |  |
| personal | Elderly people and people with disabilities have limited access to telehealth services due to poor internet skills. |  |  |  |  |  |
|  | Providers do not have sufficient health literacy to use telehealth |  |  |  |  |  |
|  | Patients do not have sufficient health literacy to use telehealth |  |  |  |  |  |
