# Supplementary material for: Barriers to widespread adoption of telehealth from physicians’ perspective: A survey in southern Iran
Source: PLoS One. 2025 Jul 21;20(7):e0327095. doi: 10.1371/journal.pone.0327095 (PMC12279122; doi:10.1371/journal.pone.0327095)
Supplement: Attachment — Submitted filename: Response_to_Editor_and_Reviewers_Comments_auresp_2.docx [file pone.0327095.s005.docx]

**Dear editor and reviewers of PLOS ONE**

We would like to express our sincere gratitude for the scientific and constructive approach adopted during the review process. We have invested a considerable amount of time and effort to address the comments made, and it is our belief that the manuscript has undergone significant enhancement in quality as a result.

The following table presents the responses to the comments made by the esteemed editor and reviewers.

| **Editor and reviewers’ comments** | **Response to Comments** |
| --- | --- |
| **Reviewer #3** | |
| **- Firstly,** referencing should happen at the end of phrases, not in the middle of the text. This is easily corrected. | **Response:** Most of the references cited have been revised. A few references have been added in the middle of text to better reflect the referred subject. |
| **-Secondly,** the Editor comments on the availability of the appendix data, your answer was: ”All the required data is within the article. There is no need for the referenced appendix.”. In all honesty, I do not think this is a good enough answer; if there was an appendix, it should be made available. In my opinion, knowing that a questionnaire was used, it should be available to readers. | **Response:** We agree with the reviewer and the questionnaire used is attached accordingly. |
| **- Thirdly,** I don’t think “adoption” should be used as a keyword for your work. I noticed the other Reviewers did not comment on it, but I suggest you evaluate the need for this word. | **Response:** We agree with the reviewer and we delete the adoption from keywords section. |
| **- Fourth**: in my opinion, the paragraph “Data was collected using electronic self-administered questionnaire. After (…) a reminder message was sent to participants twice, with an interval of one week between each reminder”, should be in the “Data col-lection” part, not in ethics | **Response:** We thank the reviewer for his/her careful comments. The cited sentences have been moved to the relevant sections. |
| **Lastly**, I think your English is very good and the paper is well structured; however, some segments are long and appear repetitive. I believe you could summarize those segments better and you work could be more concise. | **Response:** The mentioned sections are summarized as follows: |
| In order to establish the foundations for the widespread adoption of telehealth in Iran, it is first necessary to identify the barriers that currently exist. A study was conducted that reported on various barriers to the use of telecardiology, including organizational, professional and equipment-related issues [30]. Another study reported on the opinions of patients regarding virtual examinations, the inappropriateness of virtual visits for physical examinations, and the lack of patient literacy as the main barriers to tele-urology in Iran [31]. Nevertheless, hospitals in Iran have not yet met the standards required for the implementation of the telehealth network [32]. Furthermore, Iran has adopted the minimum standards for telehealth, which are less comprehensive than those of other countries. These standards are inadequate for enhancing performance and service quality [22]. Furthermore, the considerable financial and data volume costs, in addition to the technical challenges associated with their transmission, have resulted in the utilization of telehealth capabilities being largely confined to a narrow range of locations and contexts [24]. | Identifying existing barriers is a prerequisite for widespread adoption of telehealth. In Iran, Bijani et al. [30] identified organizational, professional, and equipment-related factors, while Hosseini et al. [31] identified the inappropriateness of virtual visits for physical examinations and lack of patient literacy as barriers to telehealth use.  Hospitals in Iran have not met the necessary standards for setting up a telehealth network [32]. The country has adopted minimum standards for telehealth, which are not as thorough as those in other countries. These standards do not improve performance or service quality [22]. Additionally, high costs and technical challenges related to data transmission have limited the use of telehealth services to very few places and situations [24]. |
| Despite the ongoing advancement of telecommunication technologies, evolving reimbursement mechanisms and an increasing societal inclination towards telehealth adoption (27, 28), the extensive integration of telehealth into healthcare systems is contingent upon the identification and removal of inherent obstacles. | Despite advances in telecommunication technologies, evolving reimbursement mechanisms, and growing societal acceptance of telehealth, its full integration into healthcare systems depends on removing obstacles [36, 37]. |
| The Hormozgan province's hot, humid climate, high latitude, and scattered islands in the Persian Gulf pose significant challenges to providing health services. | The heat and humidity, and the dispersion of cities and villages in Hormozgan Province, pose challenges in providing healthcare services to patients. |
| **Reviewer #4** | |
| 1. The authors didnot explicitly list the weaknesses of the designs, the barriers would have been assessed better with a mixed methods design- the qualitative methods would have given indepth responses to the barriers. | We thank the reviewer for her scientific and constructive comments and for taking the time to review the article.  **Response**: We agree with the reviewer and accordingly the following sentence has been added to the limitations section.  This study is quantitative and cross-sectional, meaning it has limits in showing cause-and-effect relationships. The self-reported data could also be influenced by social desirability bias, where participants may answer in a way they think looks good. Future studies could use qualitative or mixed methods to better understand the barriers to telehealth use in Iran. |
| 2. There is no mention on non-response bias, there were over 200 participants and only 160 responded to the survey, did the authors assess why the others didnot respond.  **Response**: Despite reminders to complete the distributed questionnaires, we did not investigate the reason for physicians' non-participation, because from a research perspective, participants were allowed to opt out of the study. | **Response**: We agree with the reviewer and accordingly we add a limitation in this regard in the limitation section as follow:  Secondly, although most physicians participated in this study, we were unable to obtain responses and perceptions from some of them regarding their use of telehealth services. Therefore, our results may be affected by non-response bias. |
