# Supplementary material for: Barriers to widespread adoption of telehealth from physicians’ perspective: A survey in southern Iran
Source: PLoS One. 2025 Jul 21;20(7):e0327095. doi: 10.1371/journal.pone.0327095 (PMC12279122; doi:10.1371/journal.pone.0327095)
Supplement: Attachment — Submitted filename: Reviewer Comments MM.docx [file pone.0327095.s002.docx]

I have been invited to review the manuscript entitled 'Barriers to Widespread Adoption of Telemedicine from Physicians’ Perspective: A Survey in Southern Iran,' which was submitted to *PLOS One* with Manuscript Number: PONE-D-24-58379. The study focuses on identifying barriers to telemedicine adoption among physicians in southern Iran. This topic is particularly important given the growing relevance of telemedicine, especially in the post-pandemic era.

My general suggestions and comments are as follows:

# Summary of the Manuscript

**Title:**

- Barriers to Widespread Adoption of Telemedicine from Physicians’ Perspective: A Survey in Southern Iran

**Objective:**

- The study aims to identify and analyses the barriers preventing the widespread adoption of telemedicine from the perspective of physicians in three teaching hospitals affiliated with Hormozgan University of Medical Sciences (HUMS).

**Methodology:**

- A cross-sectional descriptive-analytical study was conducted in 2024.
- The entire population of 239 physicians in the teaching hospitals of HUMS was included.
- Data collection was performed using a self-developed questionnaire, categorized into seven main barriers: personal, technical, behavioural, organizational, legal, clinical, and financial.
- Data analysis was done using descriptive (mean and standard deviation) and analytical (Pearson’s and Spearman’s correlation coefficients) statistical methods.

**Results:**

- 169 physicians responded.
- The highest barrier scores were for personal (3.83), technical (3.81), and behavioural (3.75) factors.
- Financial (3.54) and clinical (3.59) factors were the least concerning.
- Organizational barriers showed the highest correlation with other factors, indicating its central role in telemedicine adoption.

**Discussion:**

- Personal barriers: Limited access, low digital literacy, and lack of technical staff.
- Technical barriers: Poor infrastructure and internet connectivity issues.
- Behavioural barriers: Physician resistance, cultural beliefs, and low awareness.
- Organizational barriers: Lack of training, staff support, and national telemedicine strategies.
- Legal barriers: Concerns about patient confidentiality, data security, and malpractice.
- Clinical barriers: Lack of standard clinical protocols and challenges in physical examination.
- Financial barriers: Limited funding and unclear reimbursement policies.
- Study Strengths and Limitations:

**Strengths:**

- Comprehensive statistical analysis and identification of multiple barriers.

**Limitations:**

- Conducted in a single region, limiting generalizability.
- Incomplete participation from all physicians.

**Conclusion:**

- The study highlights the multifactorial nature of barriers to telemedicine adoption, emphasizing the need for physician training, better infrastructure, legal safeguards, and updated reimbursement policies to enhance telemedicine implementation.

# Corrections, Comments, and Suggestions

Below is an assessment of different sections of the manuscript:

# 1. Introduction

### Strengths:

- Provides a clear background on the importance of telemedicine.
- Discusses the challenges specific to Iran.

### Suggested Corrections & Improvements:

- The transition to the research objective could be clearer. The last paragraph should explicitly state how the study addresses the gap in research.
- The importance of the study should be framed in a way that highlights how it contributes to policy recommendations or future implementation strategies.

# 2. Methods

### Strengths:

- Clearly describes the study design, setting, and population.
- Provides details on questionnaire development, validation, and reliability testing.

### Suggested Corrections & Improvements:

- The ethical approval section should explicitly mention whether written informed consent was obtained from participants.
- The statistical analysis section could benefit from a more detailed explanation of why specific tests (Pearson’s & Spearman’s correlation) were used and how they align with the study objectives.

# 3. Results

### Strengths:

- Well-structured, with clear presentation of findings.
- Use of correlation analysis adds depth to understanding relationships among barriers.

### Suggested Corrections & Improvements:

- Consider adding a figure or chart to visually represent the correlations between barriers for better clarity.
- The demographic table (Table 1) should be formatted to ensure all categories are properly aligned for readability.
- Some percentages in Table 1 seem inconsistent or misplaced (e.g., "11-20 years of experience" percentage appears incorrect).

# 4. Discussion

### Strengths:

- Compares findings with prior studies from different countries.
- Provides practical recommendations for addressing barriers.

### Suggested Corrections & Improvements:

- Some points are repetitive (e.g., legal and financial barriers are discussed twice in slightly different ways). Consolidation would improve clarity.
- While discussing financial barriers, the role of insurance companies in telemedicine adoption could be further elaborated.
- The study implications should be expanded to include how policymakers and hospital administrators can implement change based on these findings.

# 5. Study Strengths and Limitations

### Strengths:

- Recognizes methodological rigor and policy implications.

### Suggested Corrections & Improvements:

- The limitations should discuss potential bias (e.g., self-reporting bias due to the questionnaire-based nature of data collection).
- A future recommendation for qualitative interviews with physicians could add depth to understanding the barriers.

# 6. Conclusion

### Strengths:

- Summarizes key findings effectively.
- Suggests actionable solutions for overcoming telemedicine barriers.

### Suggested Corrections & Improvements:

The last sentence could call for further research on solutions rather than just stating that future studies should focus on removing obstacles.

Consider mentioning specific policy changes that could enhance telemedicine adoption in Iran.

# Overall Assessment

### Strengths:

- Well-organized and methodologically sound.
- Identifies multiple dimensions of telemedicine barriers.
- Provides actionable recommendations.

### Areas for Improvement:

- Improve clarity in transitions (especially in the Introduction and Conclusion).
- Consolidate redundant discussion points.
- Add visual elements (charts or tables) to enhance readability.
- Address minor formatting and statistical explanation gaps in the Methods and Results sections.
